# La hernie diaphragmatique de Bochdalek étranglée: cause rare d’occlusion intestinale

**Published:** 2012-03-18

**Authors:** Mahmoudi Abdelhalim, Rami Mohamed, Khattala Khalid, Dahri Souad, Afifi My Abderrahmane, Bouabdallah Youssef

**Affiliations:** 1 Service de Chirurgie Pédiatrique, Hôpital Mère-Enfant, CHU Hassan II, Fès, Maroc

**Keywords:** Hernie diaphragmatique, occlusion intestinale, enfant, Maroc

## Abstract

La Hernie diaphragmatique de Bochdalek étranglée comme cause d’occlusion intestinale est très rare. Nous rapportons le cas d’un enfant de 18 mois ayant une hernie à manifestation tardive découverte au stade d’occlusion intestinale. L’enfant s’est présenté dans un tableau d’occlusion intestinale. La radiographie thoracoabdominale a montré la présence de clartés digestives en intrathoracique avec des niveaux hydroaeriques. L’exploration chirurgicale a objectivé une hernie postéro latérale gauche de Bochdalek avec étranglement du colon transverse et du grand épiploon. Le geste a consisté en la réduction des organes herniés avec fermeture du défect diaphragmatique. L’évolution est favorable avec un recul de 2 ans.

## Introduction

Les hernies diaphragmatiques congénitales (HDC) de Bochdalek de révélation tardive représentent 5 à 30% de l’ensemble des HDC. Compte tenu de l’hétérogénéité des manifestations cliniques de cette pathologie, les errances diagnostiques sont nombreuses et retardent la prise en charge. L’identification rapide des HDC de révélation tardive permet une prise en charge chirurgicale efficace avec une évolution favorable, sans séquelles, dans la majorité des cas. Nous rapportons le cas d’un enfant de 18 mois ayant une hernie à manifestation tardive découverte au stade d’occlusion intestinale qui constitue un mode de révélation particulièrement rare. L’évolution est favorable après la prise en charge spécifique.

## Patient et observation

**Figure 1:**
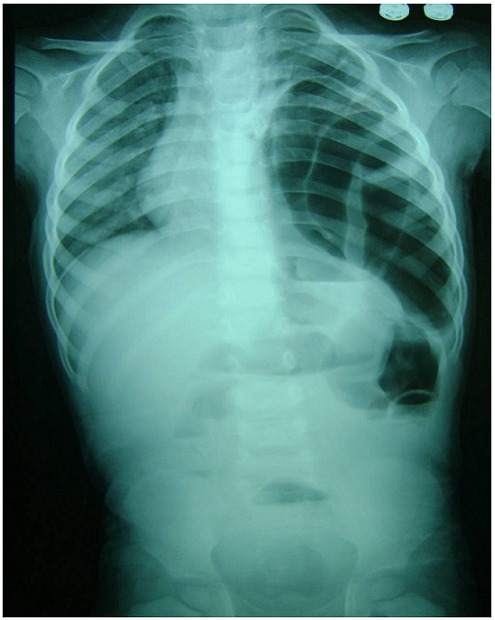
Radiographie thoracoabdominale de Face: Montrant de multiples Niveaux hydroaeriques de type grelique intrabdominaux avec des clartés digestives occupant la quasitotalité de l’hémithorax gauche faisant hernie à travers le diaphragme gauche et refoulant le médiastin à droite

**Figure 2:**
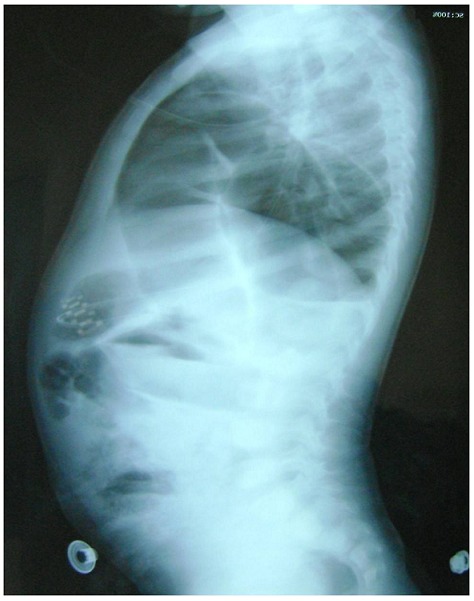
Radiographie thoracoabdominale de profil: Objective la présence de multiples NHA abdominaux avec hernie diaphragmatique de Bochdaleck à contenue colique qui occupe la quasi-totalité d’un hemichamps pulmonaire

**Figure 3:**
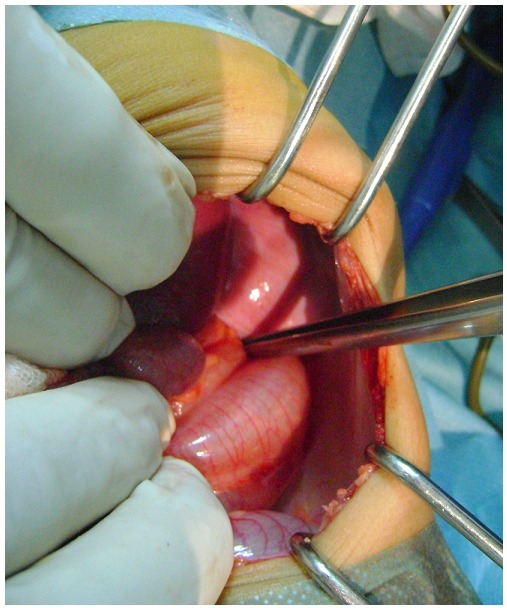
Image peropératoire objectivant une Hernie Postéro latérale gauche de Bochdaleck avec incarcération du colon transverse et du grand épiploon en intra-thoracique responsable du syndrome occlusif

Un nourrisson de 18 mois, sans antécédents particuliers. Il présente des douleurs abdominales généralisées avec des vomissements alimentaires puis bilieux depuis 3 jours avant son admission aux urgences ainsi qu’un arrêt des matières et des gaz. Il n’ y avait pas d’histoire de traumatisme. L’examen trouve un enfant apyrétique. Tachycarde à 130 battements/min, polypneique à 35 cycles/min. La saturation de l’hémoglobine en oxygène à 85% en air ambiant (98% avec un masque à oxygène et 3 l/min d’O2), le temps de recoloration cutanée supérieure à trois secondes, la pression artérielle à 95/50 mm Hg. L’examen abdominal a trouvé une distension abdominale, sans hépatomégalie ni splénomégalie, sans masse palpable, et les orifices herniaires sont libres. Les investigations biologiques étaient sans anomalies. La radiographie thoraco-abdominale trouve de multiples NHA abdominaux avec des clartés digestives dans l’hémothorax gauche, cerclées et bien délimitées, refoulant le médiastin et la trachée vers la droite. Il semblait exister une lacune pariétale diaphragmatique gauche (Figure 1, Figure 2). La mise en place d’une sonde naso-gastrique et la vidange du contenu de l’estomac amélioraient l’état de l’enfant. Après stabilisation clinique, et mesures de réanimation. Une exploration a été décidé objectivant une hernie postéro-latérale gauche avec incarcération du colon transverse et du grand épiploon en intra-thoracique (Figure 3). Une réduction des organes herniés a été réalisée suivie d’une fermeture du défect diaphragmatique de 4 cm. L’enfant est actuellement bien portant. Après un recul de 2 ans.

Le consentement du tuteur légal du patient a été obtenu. De ce fait la publication de cette observation ne pose aucun problème pour notre patient ou bien pour ses proches.

## Discussion

Les HDC de révélation tardive sont plus rares, 5 à 30% des cas [1–3]. La physiopathologie du retard d’expression de la HDC est mal connue. L’obstruction de l’orifice herniaire diaphragmatique par certains organes abdominaux tels que le foie ou la rate pourrait expliquer le délai d’apparition des signes [3].

La pathologie peut se révéler à l’occasion d’une élévation brusque de la pression abdominale (toux, effort, vomissement, traumatisme) [3]. La latence sémiologique peut être prolongée et les signes cliniques sont souvent absents jusqu’à la révélation de la hernie [1,2,4]. Les manifestations respiratoires sont prédominantes chez le petit enfant (dyspnée, wheezing, infections respiratoires à répétition, toux). Les signes sont plus volontiers de type abdominal chez l’enfant plus grand (nausées, vomissements, douleurs abdominales) [2,4,5]. Il n’existe pas de signes pathognomoniques. Des tableaux graves comme celui que nous rapportons, mettant en jeu le pronostic vital et des cas de mort subite ont été rapportés [2], mais restent rares. La quantité et la qualité des organes abdominaux ascensionnés sont variables: rate, colon, foie, estomac, rein, queue du pancréas [1,4]. Cependant, le plus souvent, colon et estomac sont les deux organes concernés [6]. Le volvulus d’un organe creux comme l’estomac, ou plein comme la rate peut être un mode de révélation [5]. Dans ces cas de HDC de révélation tardive, la localisation postéro-latérale gauche reste la plus fréquente (hernie de Bochdalek) [1].

Le diagnostic repose essentiellement sur l’examen attentif de la radiographie thoracique de face [2,6] Elle peut montrer des images de pneumopathie, de pneumothorax, d’épanchement liquidien pleural, de masses diaphragmatiques qui peuvent faire errer le diagnostic [4]. La recherche sur la radiographie thoracique, chez tout enfant présentant des symptômes respiratoires atypiques, de segments digestifs ou de niveaux hydro-aériques intra-thoraciques doit être la règle [1]. La confirmation du diagnostic peut se faire grâce à la mise en place d’une sonde œsogastrique et à l’analyse de son trajet qui pourra être intra-thoracique [1]. L’utilisation de produits de contraste aide au diagnostic [1,4]. L’échographie, la tomodensitométrie(TDM) thoracique ou l’imagerie par résonance magnétique (IRM) pulmonaire avec ingestion de produit de contraste sont utiles [1,4].

Le traitement consiste à la cure de la hernie après réduction des organes herniés soit par chirurgie classique ou coelioscopique.

## Conclusion

Les HDC de révélation tardive sont relativement rares et leur mode d’expression est divers. Le tableau d’occlusion comme mode de révélation reste particulièrement rare. Devant toute manifestation respiratoire et/ou digestive atypique, l’observation attentive de la radiographie thoracique et au moindre doute la mise en place d’une sonde œsogastrique permettent d’effectuer le diagnostic. La prise en charge chirurgicale rapide assure une évolution favorable dans la majorité des cas.
